# Plant-based diets, pescatarian diets and COVID-19 severity: a population-based case–control study in six countries

**DOI:** 10.1136/bmjnph-2021-000272

**Published:** 2021-06-07

**Authors:** Hyunju Kim, Casey M Rebholz, Sheila Hegde, Christine LaFiura, Madhunika Raghavan, John F Lloyd, Susan Cheng, Sara B Seidelmann

**Affiliations:** 1 Department of Epidemiology, Johns Hopkins Bloomberg School of Public Health, Baltimore, Maryland, USA; 2 Welch Center for Prevention, Epidemiolgy, and Clinical Research, Johns Hopkins University, Baltimore, Maryland, USA; 3 Division of Cardiovascular Medicine, Brigham & Women’s Hospital, Boston, Massachusetts, USA; 4 Envision Health Partners, Riverside, Connecticut, USA; 5 Department of Cardiology, Smidt Heart Institute, Cedars-Sinai Medical Center, Los Angeles, California, USA; 6 Department of Medicine, Stamford Hospital, Stamford, Connecticut, USA; 7 Department of Medicine, Columbia University Vagelos College of Physicians and Surgeons, New York, New York, USA

**Keywords:** COVID-19, dietary patterns

## Abstract

**Background:**

Several studies have hypothesised that dietary habits may play an important role in COVID-19 infection, severity of symptoms, and duration of illness. However, no previous studies have investigated the association between dietary patterns and COVID-19.

**Methods:**

Healthcare workers (HCWs) from six countries (France, Germany, Italy, Spain, UK, USA) with substantial exposure to COVID-19 patients completed a web-based survey from 17 July to 25 September 2020. Participants provided information on demographic characteristics, dietary information, and COVID-19 outcomes. We used multivariable logistic regression models to evaluate the association between self-reported diets and COVID-19 infection, severity, and duration.

**Results:**

There were 568 COVID-19 cases and 2316 controls. Among the 568 cases, 138 individuals had moderate-to-severe COVID-19 severity whereas 430 individuals had very mild to mild COVID-19 severity. After adjusting for important confounders, participants who reported following ‘plant-based diets’ and ‘plant-based diets or pescatarian diets’ had 73% (OR 0.27, 95% CI 0.10 to 0.81) and 59% (OR 0.41, 95% CI 0.17 to 0.99) lower odds of moderate-to-severe COVID-19 severity, respectively, compared with participants who did not follow these diets. Compared with participants who reported following ‘plant-based diets’, those who reported following ‘low carbohydrate, high protein diets’ had greater odds of moderate-to-severe COVID-19 (OR 3.86, 95% CI 1.13 to 13.24). No association was observed between self-reported diets and COVID-19 infection or duration.

**Conclusion:**

In six countries, plant-based diets or pescatarian diets were associated with lower odds of moderate-to-severe COVID-19. These dietary patterns may be considered for protection against severe COVID-19.

**What this paper adds**

- In 2884 front-line healthcare workers from six countries (France, Germany, Italy, Spain, UK, USA), individuals who reported following plant-based diets and plant-based diets or pescatarian diets that were higher in vegetables, legumes and nuts, and lower in poultry and red and processed meats, had 73% and 59% lower odds of moderate-to-severe COVID-19, respectively.
- Plant-based diets or pescatarian diets are healthy dietary patterns, which may be considered for protection against severe COVID-19.

## Introduction

Acute respiratory tract infections are a major driver of mortality and morbidity worldwide, as demonstrated by the recent coronavirus disease 2019 (COVID-19) and seasonal influenza epidemics. Globally, acute respiratory tract illnesses were estimated to cause approximately 2.4 million deaths, in people of all ages, in 2016.^1^ COVID-19 is a respiratory tract illness caused by the novel coronavirus, SARS-CoV-2, that was declared a pandemic by the WHO on 11 March 2020. Since then, several new variants of SARS-CoV-2 have emerged,^2^ adding to the global burden of infection despite public health practices including personal protective equipment (PPE), social distancing, and hand-washing. Healthcare workers (HCWs) who treat patients with COVID-19 illness in medical clinics, emergency rooms, and hospitals are particularly susceptible to contracting the infection given their high rates of exposure.^3^ While HCWs are being vaccinated in many countries currently, with the emergence of new variants and challenges in accessing COVID-19 vaccines globally, understanding risk factors associated with COVID-19 susceptibility and disease course in physicians and nurses may help to develop supportive strategies for protecting these workers both now and in the future.

Prior studies suggest a strong connection between non-hygiene-related risk factors in conferring viral disease susceptibility. Specifically, nutritional factors play a key role in both innate and adaptive immunity.^4^ Further, we have learnt that individuals with comorbidities are disproportionally affected with severe COVID-19 disease and mortality. Obesity, type 2 diabetes, atherosclerotic cardiovascular disease, and hypertension are risk factors for severe COVID-19.^5 6^ The aetiology of these conditions is largely driven by poor nutrition and unfavourable lifestyle choices (eg, physical inactivity or sedentary behaviour) which have a high prevalence in economically advantaged countries, like the USA and Europe. Yet, specific nutritional strategies to support optimal immune function have not been clearly described.

Understanding the associations between dietary patterns and COVID-19 related illness could further our understanding of the role of nutrition in viral illnesses. Using data from at-risk physicians and nurses, we aimed to evaluate the association between self-reported diets and COVID-19 infection, severity, and duration of symptoms.

## Methods

### Study design and study population

We carried out a COVID-19 case–control study of frontline physicians and nurses in six countries (France, Germany, Italy, Spain, UK, USA) evaluating nutritional factors with the risk of COVID-19 infection, severity, and duration. We leveraged a global network of physicians and nurses registered in the Survey Healthcare Globus network for healthcare market research, identifying providers with high exposure and high-risk for COVID-19-like illness. Participants completed a survey between 17 July and 25 September 2020. Details on the study design and power calculation have been reported in our earlier publications.^7 8^

Briefly, we aimed to recruit participants with a high frequency of exposure to patients with COVID-19. Participants were screened for substantial exposure to patients with COVID-19, medical specialty (eg, internal medicine, emergency medicine, critical care), practice setting (eg, emergency room, intensive care unit, other hospital-based department), presence of COVID-19 symptoms, and COVID-19 test result based on PCR or antibody. Participants could not enter into the study if they had infrequent exposure to patients with COVID-19 (defined as patient encounters meeting criteria of face-to-face within 6 feet (2 m) for greater than or equal to 10 min, the aggregate for all encounters, averaging less than 5% of time on a typical shift) unless they had COVID-19 symptoms or a positive COVID-19 test result which suggests substantial exposure. Additionally, participants whose medical specialty or practice setting was not considered to be high-risk could not enter the study. The questionnaire was terminated if participants’ response on disease severity was inconsistent with their description of COVID-19 symptoms. We screened 7344 participants for eligibility, and a total of 4460 participants were not considered eligible (online supplemental figure 1). As a result, 2884 HCWs with high frequency of exposure to patients with COVID-19 were considered eligible.

10.1136/bmjnph-2021-000272.supp1 Supplementary data

Recruitment goals for the present study was 500 cases and 2500 controls (1000 participants in the USA and 400 participants in each European country). At the time when this study was designed, the prevalence of COVID-19 was much lower than more recent estimates of COVID-19 prevalence (1.8 million cumulative cases in April 2020 compared with 115 million cumulative cases globally in March 2021).^9^ As we anticipated, enrolment of cases was slower than controls, thus, enrolment of cases was prioritised.

HCWs completed a detailed web-based questionnaire of approximately 100 items. We collected information on basic demographic characteristics, past medical history, medications, lifestyle, and COVID-19 symptoms, and a 47-item food frequency questionnaire adapted from a previously validated questionnaire that allowed us to capture food groups of the respondent’s diet.^10^ The questionnaire was translated into the primary language of each country. The English version of the questionnaire has been published.^7^ The Western Institutional Review Board at Stamford Hospital reviewed the study protocol and deemed it to be exempt. Informed consent was obtained electronically before the questionnaire was administered.

### COVID-19 cases and controls

COVID-19 cases were defined as symptomatic cases (defined as answering ‘yes’ to the question: ‘Since exposure, have you personally experienced symptoms consistent with a diagnosis of COVID-19 (fever, coughing, fatigue, loss of taste or smell)?’), or asymptomatic cases (defined as a positive PCR or antibody test without COVID-19 like symptoms (fever, coughing, fatigue, loss of taste or smell)). Controls were defined as having a negative test and/or no experience of symptoms consistent with COVID-19. Based on these definitions, there were 568 cases and 2316 controls. There were 298 cases when we restricted cases to those with a positive PCR or antibody test. We used 568 cases for our main analysis, because we considered presence of symptoms to be an important criterion. At the time of the study, HCWs in Europe and the USA may not have had timely and adequate access to COVID-19 testing. Furthermore, a negative test for SARS-CoV-2 antibodies does not indicate that HCWs did not have COVID-19.

### Severity and duration of COVID-19 cases

Cases (symptomatic and asymptomatic, n=568) were asked to rate the severity of COVID-19 illness. Participants had five options: (1) Very mild: asymptomatic or nearly asymptomatic; (2) Mild: symptoms (fever <38°C (without treatment), with or without cough, no dyspnoea, no gasping, no abnormal imaging findings); (3) Moderate: (fever, respiratory symptoms, and/or imaging findings of pneumonia); (4) Severe: meet any of the following— (a) respiratory distress, respiratory rate ≥30 times/min; (b) low oxygen saturation <93% at rest; (c) partial pressure of oxygen (PaO_2_)/fraction of inspired oxygen (FiO_2_) ≤300 mm Hg; and (5) Critical: Respiratory failure needing mechanical assistance, intensive care unit admission, shock, or extra-pulmonary organ failure. This criterion was used in a prior study.^11^ No participant selected ‘critical’ severity. We dichotomized severity of cases as moderate-to-severe versus very mild to mild.

Then, participants reported the number of days they experienced symptoms of COVID-19 (‘How many days did you experience symptoms of COVID-19? Please answer from the first day that you experienced any symptoms until you were completely asymptomatic’). We considered asymptomatic individuals to have 0 days of duration of symptoms. We compared duration of cases by dichotomising COVID-19 duration as >14 days versus ≤14 days. We used 14 days, given that 14 was the median days of duration of symptoms for individuals with moderate-to-severe COVID-19.

### Self-reported dietary patterns

Participants reported if they followed any type of specific diet over all of the past year before the COVID-19 pandemic. We used 1 year to capture usual and long-term dietary intake. Participants had 11 choices: whole foods, plant-based diet; keto diet; vegetarian diet; Mediterranean diet; pescatarian diet; Palaeolithic diet; low fat diet; low carbohydrate diet; high protein diet; other; none of the above. Before analyses, we selected dietary patterns with sufficient ‘yes’ responses (‘yes’ response of at least 100 individuals). To increase precision, we analysed three dietary patterns after combining dietary patterns that are similar in terms of dietary intake. We combined ‘whole foods, plant-based’ diets and ‘vegetarian’ diets into one category (‘plant-based diets’, n=254). Then, we combined ‘whole food, plant-based’ diets, ‘vegetarian’ diets or ‘pescatarian’ diets into another category (‘plant-based diets or pescatarian diets’, n=294) to test if a spectrum of plant-based diets which include animal products are associated with COVID-19 severity. Due to the small number of cases (nine cases of moderate-to-severe COVID-19, 40 COVID-19 cases), we could not analyse pescatarian diets separately. We used plant-based diets to encompass plant-based diets and vegetarian diets, given that vegetarian diets are considered a subset of plant-based diets which minimise consumption of animal products (meat, fish, dairy).^12 13^ Lastly, we combined ‘low carbohydrate’ diets and ‘high protein’ diets into another category (‘low carbohydrate, high protein diet’, n=483) to evaluate whether these dietary patterns are associated with COVID-19 severity. To study intake of food groups among people who reported following these dietary patterns, we categorised items in the food frequency questionnaire into 22 food groups based on nutrient composition and culinary similarities (online supplemental table 1).

### Statistical analyses

We summarised characteristics of the overall study population by severity status using mean and SD for continuous variables and percent and frequencies for categorical variables. Then, we examined food group intakes among individuals following and not following three different dietary patterns (‘plant-based diets’, ‘plant-based diets or pescatarian diets’, and ‘low carbohydrate, high protein diets’). To compare differences, we used χ^2^ test for categorical variables and analysis of variance for continuous variables.

For the primary analyses, we evaluated the association between self-reported diets and severity of COVID-19 using three multivariable logistic regression models. Model 1 adjusted for age, sex, race/ethnicity, and country. Model 2 adjusted for covariates in model 1 as well as medical specialty, smoking status, and physical activity (ORs and 95% CIs from this model were the primary results). Model 3 for adjusted covariates in model 2 as well as body mass index (BMI) and the presence of a medical condition (diagnosis of any of the following conditions: diabetes, pre-diabetes, high cholesterol, hypertension, coronary heart disease or heart attack, heart failure, cancer, prior lung disease, prior lung infection, overweight, asthma, or autoimmune disease). As secondary analyses, we used the same set of models to compare whether following ‘plant-based diets’ (reference) versus ‘low carbohydrate, high protein diets’ or ‘plant-based diets or pescatarian diets’ (reference) versus ‘low carbohydrate, high protein diets’ is associated with COVID-19 severity, and investigated the association between self-reported diets and COVID-19 infection and duration of COVID-19 illness

As a sensitivity analysis, we additionally adjusted for access to PPE, and additionally adjusted for dietary supplement use (multivitamin/mineral, folic acid/folate, vitamin A, vitamin B complex, vitamin E, zinc, N-acetyl-cysteine, choline) one at a time and simultaneously in the fully adjusted models. Dietary supplement use was defined as use of single supplements more than once per week for the previous 12 months. Although the study was underpowered to study the associations between self-reported diets and cases defined solely by testing, we limited cases to those with a positive PCR or antibody test, and repeated our analyses. All analyses were conducted using Stata version 15 statistical software (StataCorp, College Station, Texas, USA).

## Results

More than 70% of study participants were men and almost 95% of the study participants were physicians (table 1). It is possible that our study population was high in men because they may be more likely to be in a predetermined high-risk medical specialty and practice setting. Of 568 cases, 430 participants had very mild to mild severity and 138 had moderate-to-severe COVID-19 severity. HCWs in the USA, UK, Italy, and France were more likely to be moderate-to-severe cases than very mild to mild cases. Many demographic characteristics (age, sex, race/ethnicity), medical specialty, medical history, smoking status, and BMI did not significantly differ by severity of COVID-19. HCWs with moderate-to-severe COVID-19 severity were less likely to report that they followed plant-based diets (8.6% for very mild to mild cases vs 2.9% for moderate-to-severe cases; p=0.02) but were more likely to report that they followed low carbohydrate, high protein diets (table 1, 14.2% for very mild to mild cases vs 21.7% for moderate-to-severe cases; p=0.04).

**Table 1:** Characteristics of healthcare workers exposed to COVID-19 by severity status*

	Total	Controls	Cases		P value†
			Very mild to mild	Moderate to severe	
**Sample size, N**	2884	2316	430	138	
**Gender**					
Women	794 (27.5%)	640 (27.6%)	119 (27.7%)	35 (25.4%)	0.10
Men	2066 (71.6%)	1656 (71.5%)	310 (72.1%)	100 (72.5%)	
Other	2 (0.1%)	1 (0.04%)	0 (0.0%)	1 (0.7%)	
Prefer not to say	22 (0.8%)	19 (0.8%)	1 (0.2%)	2 (1.4%)	
**Age, years**	48 (10)	48 (10)	47.1 (9.5)	48.3 (9.5)	0.19
**Country**					0.048
USA	1061 (36.8%)	901 (38.9%)	116 (27.0%)	44 (31.9%)	
UK	327 (11.3%)	233 (10.1%)	66 (15.3%)	28 (20.3%)	
Spain	528 (18.3%)	382 (16.5%)	125 (29.1%)	21 (15.2%)	
Italy	433 (15.0%)	359 (15.5%)	54 (12.6%)	20 (14.5%)	
Germany	279 (9.7%)	233 (10.1%)	35 (8.1%)	11 (8.0%)	
France	256 (8.9%)	208 (9.0%)	34 (7.9%)	14 (10.1%)	
**Race/ethnicity**					
White	2218 (77.0%)	1792 (77.4%)	334 (77.7%)	92 (66.7%)	0.13
Any mixed/multiple ethnic background	162 (6.0%)	121 (5.2%)	28 (6.5%)	13 (9.4%)	
Asian	336 (12.0%)	271 (11.7%)	46 (10.7%)	19 (13.8%)	
African	48 (2.0%)	36 (1.6%)	7 (1.6%)	5 (3.6%)	
Other	36 (1.0%)	29 (1.3%)	5 (1.2%)	2 (1.4%)	
Prefer not to say	84 (3.0%)	67 (2.9%)	10 (2.3%)	7 (5.1%)	
**Job classification**					
Physician	2735 (94.8%)	2187 (94.4%)	412 (95.8%)	136 (98.6%)	0.13
Nurse/NP/PA	149 (5.2%)	129 (5.6%)	18 (4.2%)	2 (1.4%)	
**Physician specialty**					
Other	12 (0.4%)	10 (0.4%)	1 (0.2%)	1 (0.7%)	0.27
Allergy and Immunology	29 (1.0%)	25 (1.1%)	4 (0.9%)	0 (0.0%)	
Cardiology	281 (9.7%)	227 (9.8%)	38 (8.8%)	16 (11.6%)	
Critical care	282 (9.8%)	230 (9.9%)	42 (9.8%)	10 (7.2%)	
Emergency medicine	603 (20.9%)	512 (22.1%)	70 (16.3%)	21 (15.2%)	
Endocrinology, diabetes, and metabolism	98 (3.4%)	74 (3.2%)	15 (3.5%)	9 (6.5%)	
Gastroenterology	94 (3.3%)	77 (3.3%)	12 (2.8%)	5 (3.6%)	
Haematology	112 (3.9%)	85 (3.7%)	18 (4.2%)	9 (6.5%)	
Infectious disease	100 (3.5%)	82 (3.5%)	14 (3.3%)	4 (2.9%)	
Internal medicine	433 (15.0%)	322 (13.9%)	84 (19.5%)	27 (19.6%)	
Nephrology	53 (1.8%)	38 (1.6%)	15 (3.5%)	0 (0.0%)	
Neurology	107 (3.7%)	82 (3.5%)	21 (4.9%)	4 (2.9%)	
Pulmonology	430 (14.9%)	354 (15.3%)	53 (12.3%)	23 (16.7%)	
Rheumatology	101 (3.5%)	69 (3.0%)	25 (5.8%)	7 (5.1%)	
**Nurse/NP/PA practice setting**					
Emergency room	24 (16.1%)	22 (17.1%)	1 (6.0%)	1 (50.0%)	0.12
Intensive care unit	50 (33.6%)	45 (34.9%)	5 (28.0%)	0 (0.0%)	
Other hospital-based department	75 (50.3%)	62 (48.1%)	12 (67.0%)	1 (50.0%)	
**COVID-19 test (PCR or antibody**)					
No – I did not get a test	748 (25.9%)	695 (30.0%)	38 (8.8%)	15 (10.9%)	<0.001
No – I did not have access to the test	101 (3.5%)	69 (2.9%)	21 (4.9%)	11 (8.0%)	
Yes – I tested negative	1737 (60.2%)	1552 (67.0%)	160 (37.2%)	25 (18.1%)	
Yes – I tested positive	298 (10.3%)	0 (0%)	211 (49.1%)	87 (63.0%)	
**Presence of pre-existing medical conditions**	1264 (44.0%)	1002 (43.0%)	196 (45.6%)	66 (47.8%)	0.65
**Smoking status**					0.64
Never smoker	2323 (80.5%)	1865 (80.5%)	344 (80.0%)	114 (82.6%)	
Former smoker	427 (14.8%)	341 (14.7%)	66 (15.3%)	20 (14.5%)	
Current smoker	134 (4.6%)	110 (4.7%)	20 (4.7%)	4 (2.9%)	
**Body mass index (kg/m^2^**)	25.0 (4.3)	24.9 (4.2)	24.9 (4.8)	25.2 (4.8)	0.57
**Self-reported diets**‡					
Plant-based diets§	254 (8.8%)	213 (9.2%)	37 (8.6%)	4 (2.9%)	0.02
Plant-based diets or pescatarian diets¶	294 (10.2%)	248 (10.7%)	40 (9.3%)	6 (4.3%)	0.06
Low carbohydrate, high protein diets**	483 (16.7%)	392 (16.9%)	61 (14.2%)	30 (21.7%)	0.04

*Values are n (%) for categorical variables and mean (SD) for continuous variables. Cases are defined as individuals with self-reported COVID-19-like illness (fever, coughing, fatigue, loss of taste or smell) or a positive PCR or antibody test.

†P value compared moderate-to-severe severity versus very mild to mild severity among cases.

‡Participants were asked to report if they followed any type of specific diet over the past 12 months before the COVID-19 pandemic.

§Participants self-reported that they followed whole foods, plant-based diets or vegetarian diets.

¶Participants self-reported that they followed whole foods, plant-based diets or vegetarian diets or pescatarian diets.

**Participants self-reported that they followed low carbohydrate diets or high protein diets.

NP, nurse practitioner; PA, physician assistant.

Among COVID-19 cases, individuals who reported following plant-based diets consumed more total vegetables, plant proteins (legumes and nuts), and less poultry, red and processed meats, sugar-sweetened beverages, and alcohol compared with those who did not follow plant-based diets (table 2). Similarly, individuals who reported following a plant-based diet or pescatarian diet consumed more vegetables, legumes and nuts and less poultry and red and processed meats compared with those who did not follow either of this dietary pattern (table 3). Qualitatively, fish and seafood intake was slightly higher among individuals who reported following a plant-based diet or pescatarian diet (3.0 times/week) than those following a plant-based diet (2.5 times per week). Fruit consumption was higher among those who followed a plant-based diet or pescatarian diet (9.9 times/week) than those who did not follow these diets (8.5 times/week), but this difference was not statistically significant (p=0.15). Participants who reported following low carbohydrate, high protein diets had higher intake of legumes, nuts, soups, and animal products (eg, eggs, poultry), and lower intake of refined grains, sweets and desserts, vegetable oil, croquettes, dumplings, and pizza compared with participants who did not follow low carbohydrate, high protein diets (online supplemental table 2).

**Table 2:** Dietary intake of healthcare workers stratified by self-report of following plant-based diet among COVID-19 cases (n=568)*

	Followed plant-based diet (n=41)	Did not follow plant-based diet (n=527)	P value
Dietary intake, times/week (mean, SD)			
Total fruits	9.8 (6.4)	8.5 (6.5)	0.23
**Total vegetables**	**14.5** (**8.7**)	**10.4** (**7.1**)	**<0.001**
Potatoes	2.1 (1.9)	2.1 (1.8)	0.90
**Legumes**	**3.7** (**2.9**)	**1.9** (**1.6**)	**<0.001**
**Nuts**	**3.5** (**2.6**)	**2.3** (**2.9**)	**0.01**
Refined grains	7.5 (5.5)	8.6 (5.2)	0.17
Dark or whole grain breads	2.5 (2.2)	2.2 (2.5)	0.55
Sweets and desserts	5.8 (5.8)	6.8 (6.9)	0.35
Eggs	2.0 (1.8)	2.3 (1.9)	0.30
Dairy	12.9 (9.1)	13.3 (7.9)	0.73
**Poultry**	**1.2** (**1.5**)	**2.3** (**1.6**)	**<0.001**
**Red and processed meats**	**1.3** (**2.3**)	**3.8** (**2.8**)	**<0.001**
Fish and seafood	2.5 (2.7)	3.1 (2.6)	0.12
Soups	1.4 (1.7)	1.4 (1.4)	0.78
Croquettes, dumplings, pizza	0.8 (0.8)	1.0 (1.0)	0.14
**Sugar-sweetened beverages**	**1.1** (**2.1**)	**2.5** (**3.4**)	**0.01**
Fruit juices	0.4 (0.9)	1.0 (1.9)	0.06
Vegetable oil	3.6 (3.3)	3.8 (3.2)	0.67
Butter	1.4 (2.0)	1.9 (2.3)	0.15
**Alcohol**	**2.2** (**3.0**)	**3.7** (**4.3**)	**0.03**
Coffee	6.5 (5.1)	7.7 (6.8)	0.27
Tea	1.9 (2.5)	2.1 (3.6)	0.68

Bold font denotes statistically significant associations.

*Cases are defined as individuals with self-reported COVID-19-like illness (fever, coughing, fatigue, loss of taste or smell) or a positive PCR or antibody test. P value comparing those following a plant-based diet versus those not following a plant-based diet among cases. Details on these food groups are presented in online supplemental table 1.

**Table 3:** Dietary intake of healthcare workers stratified by self-report of following plant-based diet or pescatarian diet among COVID-19 cases (n=568)*

	Followed plant-based diet or pescatarian diet (n=46)	Did not follow plant-based or pescatarian diet (n=522)	P value
Dietary intake, times/week (mean, SD)			
Total fruits	9.9 (6.4)	8.5 (6.5)	0.15
**Total vegetables**	**14.4** (**8.4**)	**10.4** (**7.1**)	**<0.001**
Potatoes	2.2 (1.9)	2.1 (1.7)	0.89
**Legumes**	**3.7** (**2.8**)	**1.9** (**1.6**)	**<0.001**
**Nuts**	**3.6** (**2.5**)	**2.3** (**2.9**)	**0.004**
Refined grains	7.5 (6.1)	8.6 (5.2)	0.17
Dark or whole grain breads	2.7 (2.4)	2.2 (2.5)	0.21
Sweets and desserts	6.7 (9.3)	6.7 (6.5)	0.96
Eggs	2.2 (2.1)	2.3 (1.9)	0.80
Dairy	13.4 (9.0)	13.3 (7.9)	0.90
**Poultry**	**1.3** (**1.6**)	**2.3** (**1.6**)	**<0.001**
**Red and processed meats**	**1.7** (**3.3**)	**3.8** (**2.8**)	**<0.001**
Fish and seafood	3.0 (3.9)	3.1 (2.5)	0.86
Soups	1.5 (1.7)	1.4 (1.4)	0.73
Croquettes, dumplings, pizza	0.9 (1.4)	1.0 (1.0)	0.62
Sugar-sweetened beverages	1.5 (2.6)	2.5 (3.4)	0.06
Fruit juices	0.5 (1.3)	1.0 (1.9)	0.12
Vegetable oil	3.5 (3.2)	3.8 (3.3)	0.59
Butter	1.6 (2.1)	1.9 (2.3)	0.35
Alcohol	2.6 (4.1)	3.7 (4.3)	0.09
Coffee	6.2 (5.1)	7.7 (6.8)	0.12
Tea	2.2 (2.7)	2.1 (3.6)	0.92

Bold font denotes statistically significant associations.

*Cases are defined as individuals with self-reported COVID-19 like illness (fever, coughing, fatigue, loss of taste or smell) or a positive PCR or antibody test. P value comparing those following a plant-based or pescatarian diet versus those not following a plant-based or pescatarian diet among cases. Details on these food groups are presented in online supplemental table 1.

**Figure 1:**
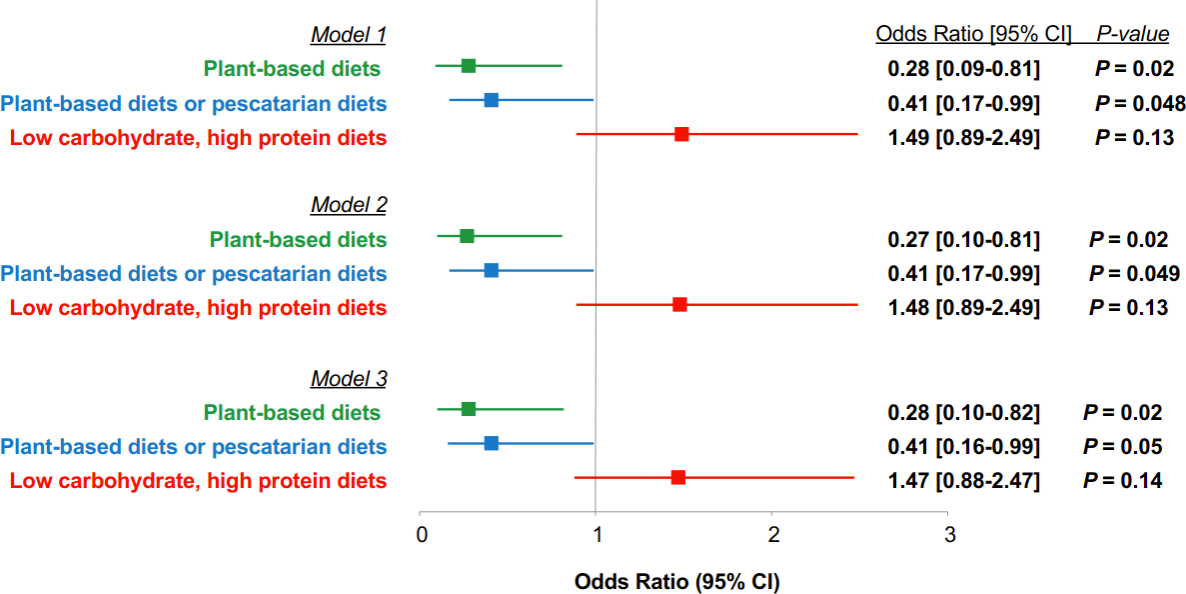
Adjusted odds ratios (ORs) and 95% confidence intervals (95% CI) for the association between self-reported dietary patterns and moderate-to-severe COVID-19. ORs of moderate-to-severe COVID-19 for those who followed low carbohydrate, high protein diets were 3.55 (95% CI 1.06 to 11.82) in model 1, 3.86 (95% CI 1.13 to 13.24) in model 2, and 3.96 (95% CI 1.14 to 13.75) in model 3 (p<0.05 for all tests), compared with those who followed plant-based diets. ORs for moderate-to-severe COVID-19 for those who followed low carbohydrate, high protein diets were 2.36 (95% CI 0.83 to 6.71) in model 1, 2.51 (95% CI 0.87 to 7.26) in model 2, and 2.60 (95% CI 0.88 to 7.66) in model 3 (p>0.05 for all tests), compared with those who followed plant-based diets or pescatarian diets. We compared moderate-to-severe severity to very mild to mild severity. ‘Very mild’ severity was defined as asymptomatic or nearly asymptomatic. ‘Mild’ severity was defined as symptoms (fever <38°C (without treatment), with or without cough, no dyspnoea, no gasping, no abnormal imaging findings). ‘Moderate’ severity was defined as fever, respiratory symptoms, and/or imaging findings of pneumonia. ‘Severe’ severity was defined as meeting any of the following: (1) respiratory distress, respiratory rate ≥30 times/min; (2) low oxygen saturation (SpO_2_) <93% at rest; (3) partial pressure of oxygen (PaO_2_)/fraction of inspired oxygen (FiO_2_) ≤300 mm Hg. Model 1 adjusted for age, sex, race/ethnicity, and country. Model 2 additionally adjusted for specialty, smoking, and physical activity. Model 3 additionally adjusted for body mass index and presence of a medical condition.

After adjusting for basic demographic characteristics, medical specialty, and health behaviours (smoking, physical activity) in model 2, participants who followed plant-based diets had 73% lower odds of moderate-to-severe COVID-19 (OR 0.27, 95% CI 0.10 to 0.81) compared with participants who did not follow plant-based diets (figure 1). Similarly, participants who followed either plant-based diets or pescatarian diets had 59% lower odds of moderate-to-severe COVID-19 (OR 0.41, 95% CI 0.17 to 0.99) compared with those who did not follow these diets. These associations did not change when BMI and the presence of a medical condition was further adjusted. Compared with those who did not follow low carbohydrate, high protein diets, following these diets was associated with 48% greater odds of moderate-to-severe COVID-19 (OR 1.48, 95% CI 0.89 to 2.49) in a model adjusting for demographic characteristics, medical specialty, and health behaviours. However, the association between low carbohydrate, high protein dietary pattern was not statistically significant in model 1 (p=0.13), model 2 (p=0.13), or model 3 (p=0.14).

We then changed comparisons to those who reported following plant-based diets to those who reported following low carbohydrate, high protein diets. Compared with those who followed plant-based diets, those who followed low carbohydrate, high protein diets had >3-fold higher odds of moderate-to-severe COVID-19 (OR for model 1: 3.55, 95% CI 1.06 to 11.82; OR for model 2: 3.86, 95% CI 1.13 to 13.24; OR for model 3: 3.96, 95% CI 1.14 to 13.75, p<0.05 for all tests). Compared with those who followed plant-based diets or pescatarian diets, those who followed low carbohydrate, high protein diets had non-significant greater odds of moderate-to-severe COVID-19 (OR for model 1: 2.36, 95% CI 0.83 to 6.71; OR for model 2: 2.51, 95% CI 0.87 to 7.26; OR for model 3: 2.60, 95% CI 0.88 to 7.66, p>0.05 for all tests). No significant association was observed between any of the self-reported diets and odds of COVID-19 illness (online supplemental table 3; p values for all tests >0.05) or duration of COVID-19 (online supplemental table 4; p values for all tests >0.05).

In a sensitivity analysis, the association did not change substantially when we additionally adjusted for access to PPE for plant-based diets (OR 0.29, 95% CI 0.10 to 0.84) and for low carbohydrate, high protein diets (OR 1.48, 95% CI 0.87 to 2.52), but the confidence interval was wider and the association was no longer statistically significant for plant-based diets or pescatarian diets (OR 0.42, 95% CI 0.17 to 1.05). When we additionally adjusted for use of different dietary supplements one at a time and simultaneously, the results were unchanged from the main analyses (data not shown). When we limited cases to those with a positive PCR or antibody test, the association between self-reported diets and moderate-to-severe COVID-19 illness was less precise, but the direction of the association was consistent with the main analysis (model 2, OR for plant-based diets: 0.49, 95% CI 0.13 to 1.86; OR for plant-based diets or pescatarian diets: 0.47, 95% CI 0.12 to 1.78; OR for low carbohydrate, high protein diets: 1.31, 95% CI 0.62 to 2.76) (online supplemental table 5). In this sensitivity analysis limited to cases with a positive PCR or antibody test, we observed no significant association between self-reported diets and odds of COVID-19 illness or duration of COVID-19, except for plant-based diets or pescatarian diets and lower odds of COVID-19 illness (model 2, OR 0.56, 95% CI 0.34 to 0.95).

## Discussion

In HCWs from six countries with a high frequency of exposure to COVID-19 patients, following plant-based diets or a spectrum of plant-based diets (plant-based diets or pescatarian diets) was associated with 73% and 59% lower odds of moderate-to-severe COVID-19-like illness, respectively, compared with individuals who did not follow these diets. Following low carbohydrate, high protein diets was associated with a non-significant 48% greater odds of moderate-to-severe COVID-19-like illness, compared with individuals who did not follow these diets. However, compared with those who reported following plant-based diets, those who followed low carbohydrate, high protein diets had higher odds of moderate-to-severe COVID-19. No association was observed for self-reported diets and odds of COVID-19-like illness or duration of COVID-19 symptoms.

To our knowledge, this is the first study to report an association between dietary patterns and severity of COVID-19 illness. Several studies have hypothesised that healthy dietary patterns may play a role in the incidence or disease course of COVID-19 by improving the immune response.^14–16^ Despite the growing interest in identifying nutritional strategies that may mitigate the risk of COVID-19 infection or patient outcomes, only one previous study examined dietary habits and COVID-19 severity. In a single-centre, cross-sectional study of 206 Iranian patients with COVID-19, those with higher fruit and poultry consumption and lower tea consumption were less likely to have severe COVID-19.^17^ Our findings were similar to this prior study in that participants who reported following plant-based diets or a spectrum of plant-based diets (plant-based diets or pescatarian diets) had a non-significant higher intake of fruits. However, in our study, participants who were following plant-based diets or pescatarian diets had higher consumption of other food groups (vegetables, legumes, nuts) and lower consumption of poultry. Such differences in results may be due to differences in dietary patterns among Iranian adults relative to our study population, and our focus on dietary patterns rather than intake of specific food groups.

Plant-based diets or vegetarian diets are dietary patterns that are high in plant foods and low in animal products.^12 13^ Consistent with this definition, participants who reported that they followed plant-based diets or vegetarian diets had higher intake of plant foods (vegetables, legumes, nuts) and lower intake of poultry and red and processed meat. Plant-based diets are rich in nutrients, especially phytochemicals (polyphenols, carotenoids),^13 18^ with prior studies reporting higher fibre, vitamins A, C, and E, folate, and mineral (iron, potassium, magnesium) intake among those with highest versus lowest adherence to plant-based diets.^12 19^ Studies have reported that supplementation of some of these nutrients, specifically, vitamins A, C, D, and E, decreased the risk of respiratory infections, such as the common cold and pneumonia, and shortened the duration of these illnesses.^16 20–23^ These nutrients are hypothesised to support the immune system as they play important roles in the production of antibodies, proliferation of lymphocytes, and reduction of oxidative stress.^16^ While previously, both specific micronutrient deficiencies as well as generalised malnutrition have been associated with immune dysfunction in the host, the viral pathogen itself may be affected by the nutritional deficiency as well.^4 24^ Multiple viruses, such as coxsackievirus and influenza, have been shown to develop increased virulence due to changes in their genomes as a consequence of replicating in a nutritionally deficient host (eg, deficiency in selenium).^25 26^ Selenium may be an important nutrient to consider, considering its role in immunity.^16 27^ Given our findings of protection from severe illness in those consuming a micronutrient-dense diet, our data support this hypothesis. We could not further explore individual nutrient levels in our study population because we did not have these data. Future studies with detailed information on plasma micronutrient levels are warranted to confirm our findings.

Along with plant-based diets, individuals who reported following pescatarian diets had lower odds of severe COVID-19. Pescatarian diets lie within the spectrum of plant-based diets and include fish or seafood while restricting the intake of meats.^28^ Fish intake is an important source of vitamin D and omega-3 fatty acids, that is, eicosapentaenoic acid (EPA) and docosahexaenoic acid (DHA). High EPA and DHA intake, which results in high omega-3 fatty acids compared with omega-6 fatty acids and formation of omega-3 oxylipins,^29^ have anti-inflammatory effects, suppressing the production of pro-inflammatory cytokines (interleukin 1β, tumour necrosis factor α), reducing inflammatory eicosanoid synthesis and oxidative stress.^15 16 30^ A meta-analysis of 10 randomised controlled trials in patients with acute respiratory distress syndrome (ARDS) found that individuals who received formulas high in omega-3 fatty acid had shorter duration of mechanical ventilation and shorter length of stay at intensive care units,^30^ suggesting favourable effects of omega-3 fatty acids on ARDS. In our study, the magnitude of the association for moderate-to-severe COVID-19 illness was lower when we included pescatarian diets in addition to those who reported following plant-based diets. Our results suggest that although there is evidence that fish intake may have favourable impacts on respiratory illness, future studies are needed to confirm whether pescatarian diets are associated with patient outcomes in the context of COVID-19.

Interestingly, when we restricted cases to those with a positive PCR or antibody test, individuals who reported following plant-based diets or pescatarian diets had lower odds of COVID-19 infection. Epidemiological studies have shown that fruit and vegetable intake is associated with a lower risk of upper respiratory tract infection such as cold, influenza, or sinusitis.^31^ Some studies reported that higher intake of fish was inversely associated with respiratory conditions such as asthma and chronic obstructive pulmonary disease.^32 33^ However, considering the lack of data on dietary intake and COVID-19 in the literature, and the smaller number of cases when we defined cases solely by testing, the result on COVID-19 infection requires replication.

Low carbohydrate, high protein diets were associated with non-significant greater odds of severe COVID-19 when compared with those who did not report following these diets. However, when we compared these diets to plant-based diets, those who reported following low carbohydrate, high protein diets had significantly greater odds of moderate-to-severe COVID-19. Participants who reported following low carbohydrate, high protein diets had a higher intake of legumes, nuts, soups, and animal products such as eggs and poultry. Studies have hypothesised that an unhealthy dietary pattern, such as Western diets which are high in refined sugars, processed foods, and red and processed meats, can be pro-inflammatory and have negative health impacts.^15 34^ Without plasma micronutrient levels, it is unclear whether low carbohydrate, high protein diets are unhealthy. Nonetheless, our results highlight that it may be advisable to follow a healthy dietary pattern such as plant-based diets or pescatarian diets.

Our study has several strengths, including a large sample size, diverse HCWs from multiple countries, and careful adjustment of potential confounding factors. Further, we were able to capture the early phase of the global pandemic, before the detection of known SARS-CoV2 variants of concern, which could further complicate a multinational epidemiological analysis, by leveraging an existing network of HCWs. However, our finding should be interpreted within the context of the following limitations. First, we relied on participants’ self-report to define exposures and outcomes. As such, there is a possibility of recall bias. However, HCWs are unique in that they may be a source of high-quality data (eg, complete information, probably high accuracy of information).^35^ Second, the definition of certain dietary patterns (eg, plant-based diets, pescatarian diets, low carbohydrate, high protein diet) may vary by countries. Nevertheless, it is encouraging that the responses on the food frequency questionnaire reflected intake of food groups that are consistent with these dietary patterns. Third, our study may not have included individuals with more severe COVID-19 illness, given that severe cases (mechanical ventilation, admission to intensive care units) may not have been able to complete our questionnaire. Fourth, our study population comprised predominantly male physicians; thus, the findings of the study may need to be replicated in women and non-HCWs. Lastly, despite our efforts to adjust for a number of confounding factors, residual confounding may still be present due to unmeasured or incorrectly assessed variables.

In conclusion, individuals who reported following plant-based diets or pescatarian diets had lower odds of severe COVID-19-like illness. Individuals who reported following low carbohydrate, high protein diets had higher odds of severe-COVID-19-like illness, when compared with individuals who followed plant-based diets. Those who reported following plant-based diets or pescatarian diets had higher intake of vegetables, legumes and nuts, and lower intake of poultry and red and processed meats. Our results suggest that a healthy diet rich in nutrient-dense foods may be considered for protection against severe COVID-19. Future studies with detailed macro- and micronutrient data are warranted to study associations between dietary intake and COVID-19 severity.

## Data Availability

Data are not publicly available.
