# Supplementary material for: Plant-based diets, pescatarian diets and COVID-19 severity: a population-based case–control study in six countries
Source: BMJ Nutr Prev Health. 2021 May 18:bmjnph-2021-000272. doi: 10.1136/bmjnph-2021-000272 (PMC8219480; doi:10.1136/bmjnph-2021-000272)

Supplemental Table 1. Classification of food items in the food frequency questionnaire to food groups[1]

Food groups	Items in the food frequency questionnaire
Total fruits	Citrus fruits such as orange, tangerine; other fruits, such as apple, pear, peach, banana; canned fruits (in syrup)
Total vegetables	Salad: lettuce, tomato, endive; green vegetables such as green beans, chard or spinach; other vegetables, such as eggplants, mushrooms
Potatoes	Baked, fried or boiled potatoes
Legumes	Lentils, chickpeas, beans
Nuts	Nuts such as peanuts, hazelnuts, almonds
Refined grains	Puffed breakfast cereals ("Corn flakes," "Kellogg's," "Miel pops", "Vitalis," "Cheerios"), Snack bags ("chips," "potato crisps," "Cheetos," "Fritos"); white rice, paella; pasta such as noodles, macaroni, spaghetti; white bread in sandwich or with meals
Dark or whole grain breads	Dark or whole grain breads
Sweets and desserts	Chocolate: table, chocolates, "Kit Kat," "Mars," or "M&M's"; treats such as jelly beans, candy; sweet cookies or crackers; cookies with chocolate, cream; cupcakes, sponge cake; chocolate or cream cakes; pastries, donuts, croissants
Eggs	Eggs
Dairy	Milk; yogurt/yoghurt; white or fresh cheese (Burgoes) or low-fat cheese; other cheeses such as cured or semi-cured, creamy; dairy desserts such as custard, flan, cottage cheese; ice creams
Poultry	Chicken or turkey
Red and processed meats	Veal, pork, lamb such as steak, patty; minced meat, longaliza, hamburger; salty, sweet ham; sausages
Fish and seafood	White fish such as hake, grouper; blue fish such as sardines, tuna, salmon; seafood such as mussels, prawns, squid
Soups	Soups or cream-based soups
Croquettes, dumplings, pizza	Croquettes, dumplings, pizza
Sugar-sweetened beverages	Sugary drinks such as "Coca-Cola," "Fanta"; low calorie drinks such as diet coke, coca-cola light
Fruit juices	Commercial fruit juices
Vegetable oil	Vegetable oil such as olive oil, corn, canola, or rapeseed oil
Butter	Butter
Alcohol	Wine, sangria; beer (not including alcohol-free beer); distilled beverages such as whiskey, gin, cognac
Coffee	Coffee
Tea	Black or green tea

[1] We used the food frequency questionnaire published in Rodríguez IT, Ballart JF, Pastor GC, Jordà EB, Val VA. [Validation of a short questionnaire on frequency of dietary intake: reproducibility and validity]. *Nutr Hosp*. 2008;**23**:242-252.

Supplemental Table 2. Dietary intake of health care workers stratified by self-report of following low carbohydrate, high protein diet among Covid-19 cases (N=568)[a]

	Followed low carbohydrate, high protein diet (n=91)	Did not follow low carbohydrate, high protein diet (n=477)	*P*-value
Dietary intake, times/week (Mean, SD)			
Total fruits	8.2 (5.7)	8.7 (6.7)	0.59
Total vegetables	11.7 (8.9)	10.5 (7.0)	0.16
Potatoes	2.2 (2.4)	2.1 (1.6)	0.68
**Legumes**	**2.4 (2.4)**	**1.9 (1.7)**	**0.02**
**Nuts**	**3.1 (4.6)**	**2.3 (2.5)**	**0.01**
**Refined grains**	**6.8 (5.6)**	**8.9 (5.1)**	**<0.001**
Dark or whole grain breads	2.1 (2.2)	2.3 (2.5)	0.67
**Sweets and desserts**	**5.2 (6.4)**	**7.0 (6.8)**	**0.02**
**Eggs**	**3.0 (2.6)**	**2.1 (1.7)**	**<0.001**
Dairy	12.0 (9.7)	13.5 (7.6)	0.09
**Poultry**	**2.7 (2.0)**	**2.2 (1.6)**	**0.008**
Red and processed meats	3.4 (3.1)	3.7 (2.8)	0.39
Fish and seafood	3.3 (3.0)	3.0 (2.6)	0.34
**Soups**	**1.7 (1.7)**	**1.3 (1.4)**	**0.04**
**Croquettes, dumplings, pizza**	**0.8 (0.9)**	**1.0 (1.0)**	**0.01**
Sugar-sweetened beverages	2.6 (4.0)	2.3 (3.2)	0.43
Fruit juices	0.9 (1.4)	0.9 (1.9)	0.92
**Vegetable oil**	**3.0 (2.7)**	**3.9 (3.3)**	**0.011**
Butter	1.7 (2.0)	1.9 (2.4)	0.43
Alcohol	3.5 (4.6)	3.6 (4.2)	0.75
Coffee	7.1 (6.8)	7.7 (6.6)	0.38
Tea	2.5 (3.5)	2.0 (3.6)	0.23

[a] Cases are defined as individuals with self-reported Covid-19-like illness (fever, coughing, fatigue, loss of taste or smell) or a positive PCR or antibody test. *P*-value comparing those following a low carbohydrate, high protein diet vs. those not following a low carbohydrate, high protein diet. Details on these food groups are presented in **Supplemental Table 1**.

Bold font denotes statistically significant associations.

Supplemental Table 3. Adjusted Odds ratios (OR) and 95% confidence intervals (CI) for the association between self-reported diets and odds of Covid-19 (N=2884)[a]

	OR (95% CI)					
	**Model 1[b]**	***P*-value**	**Model 2[c]**	***P*-value**	**Model 3[d]**	***P*-value**
Plant-based diets	0.79 (0.56, 1.14)	0.21	0.80 (0.56, 1.14)	0.21	0.81 (0.56, 1.15)	0.24
Plant-based diets or pescatarian diets	0.76 (0.54, 1.07)	0.11	0.76 (0.55, 1.07)	0.12	0.77 (0.55, 1.08)	0.14
Low carbohydrate, high protein diets	1.01 (0.78, 1.31)	0.94	1.01 (0.78, 1.31)	0.92	1.00 (0.78, 1.30)	0.98

[a] Cases are defined as individuals with self-reported Covid-19-like illness (fever, coughing, fatigue, loss of taste or smell) or a positive PCR or antibody test.
[b] Model 1 adjusted for age, sex, race/ethnicity, and country.
[c] Model 2 additionally adjusted for specialty, smoking, and physical activity
[d] Model 3 additionally adjusted for body mass index and presence of a medical condition

Supplemental Table 4. Adjusted Odds ratios (OR) and 95% confidence intervals (CI) for the association between self-reported diets and duration of Covid-19 among Covid-19 cases (N=568)[a]

	OR (95% CI)					
	**Model 1[b]**	***P*-value**	**Model 2[c]**	***P*-value**	**Model 3[d]**	***P*-value**
Plant-based diets	1.19 (0.53, 2.66)	0.67	1.21 (0.54, 2.71)	0.64	1.25 (0.56, 2.82)	0.58
Plant-based diets or pescatarian diets	1.15 (0.54, 2.48)	0.71	1.17 (0.54, 2.52)	0.69	1.21 (0.56, 2.62)	0.63
Low carbohydrate, high protein diets	1.22 (0.68, 2.19)	0.51	1.19 (0.66, 2.15)	0.57	1.17 (0.65, 2.13)	0.56

[a] Cases are defined as individuals with self-reported Covid-19-like illness (fever, coughing, fatigue, loss of taste or smell) or a positive PCR or antibody test. We compared >14 days vs. ≤14 days. Participants reported the number of days they experienced symptoms of Covid-19. Asymptomatic individuals with a positive PCR or antibody test were considered to have 0 days of Covid-19 symptom duration.
[b] Model 1 adjusted for age, sex, race/ethnicity, and country.
[c] Model 2 additionally adjusted for specialty, smoking, and physical activity
[d] Model 3 additionally adjusted for body mass index and presence of a medical condition

Supplemental Table 5. Adjusted Odds ratios (OR) and 95% confidence intervals (CI) for the association between self-reported diets and odds of Covid-19-positive test (by PCR or antibody), severity, and duration of Covid-19[a]

	**OR (95% CI)**					
	**Model 1[b]**	***P*-value**	**Model 2[c]**	***P*-value**	**Model 3[d]**	***P*-value**
**Covid-19 test**						
Plant-based diets	0.62 (0.36, 1.05)	0.08	0.62 (0.37, 1.07)	0.09	0.62 (0.37, 1.07)	0.09
Plant-based diets or pescatarian diets	**0.55 (0.33, 0.93)**	**0.02**	**0.56 (0.34, 0.95)**	**0.03**	**0.57 (0.34, 0.95)**	**0.03**
Low carbohydrate, high protein diets	0.89 (0.62, 1.27)	0.51	0.90 (0.63, 1.29)	0.56	0.90 (0.63, 1.28)	0.56
**Moderate-to-severe vs. mild to very mild severity**						
Plant-based diets	0.50 (0.13, 1.90)	0.31	0.49 (0.13, 1.86)	0.29	0.48 (0.13, 1.86)	0.29
Plant-based diets or pescatarian diets	0.48 (0.13, 1.81)	0.28	0.47 (0.12, 1.78)	0.26	0.46 (0.12, 1.77)	0.26
Low carbohydrate, high protein diets	1.29 (0.61, 2.72)	0.51	1.31 (0.62, 2.76)	0.48	1.31 (0.62, 2.77)	0.48
**>14 days vs. ≤14 days of Covid-19 symptoms**						
Plant-based diets	0.74 (0.19, 2.84)	0.66	0.70 (0.18, 2.77)	0.62	0.70 (0.18, 2.74)	0.61
Plant-based diets or pescatarian diets	0.66 (0.17, 2.53)	0.55	0.64 (0.17, 2.47)	0.52	0.65 (0.17, 2.50)	0.53
Low carbohydrate, high protein diets	1.85 (0.82, 4.16)	0.14	1.86 (0.82, 4.22)	0.14	1.82 (0.80, 4.14)	0.15

[a] Cases are defined as individuals a positive PCR or antibody test (N=298 cases and N=2316 controls). Cases were asked to rate the severity of Covid-19 illness. We compared moderate-to-severe severity (N=87) to very mild to mild severity (N=211). Severity was defined as: 1) Very mild: asymptomatic or nearly asymptomatic, 2) Mild: symptoms [fever <38°C (without treatment), with or without cough, no dyspnea, no gasping, no abnormal imaging findings], 3) Moderate: [fever, respiratory symptoms, and/or imaging findings of pneumonia], 4) Severe: meet any of the following: 1) respiratory distress, respiratory rate ≥30 times/min 2) low oxygen saturation <93% at rest 3) partial pressure of oxygen (PaO2)/ fraction of inspired oxygen (FiO2) ≤ 300 mm Hg. Cases also reported the number of days they experienced symptoms of Covid-19. We compared >14 days (N=73) to ≤14 days of Covid-19 symptoms (N=225).

[b] Model 1 adjusted for age, sex, race/ethnicity, and country.

[c] Model 2 additionally adjusted for specialty, smoking, and physical activity

[d] Model 3 additionally adjusted for body mass index and presence of a medical condition

Bold font denotes statistically significant associations.

BMJ Publishing Group Limited (BMJ) disclaims all liability and responsibility arising from any reliance placed on this supplemental material which has been supplied by the author(s)

Supplemental Figure 1. Flow diagram of study participants

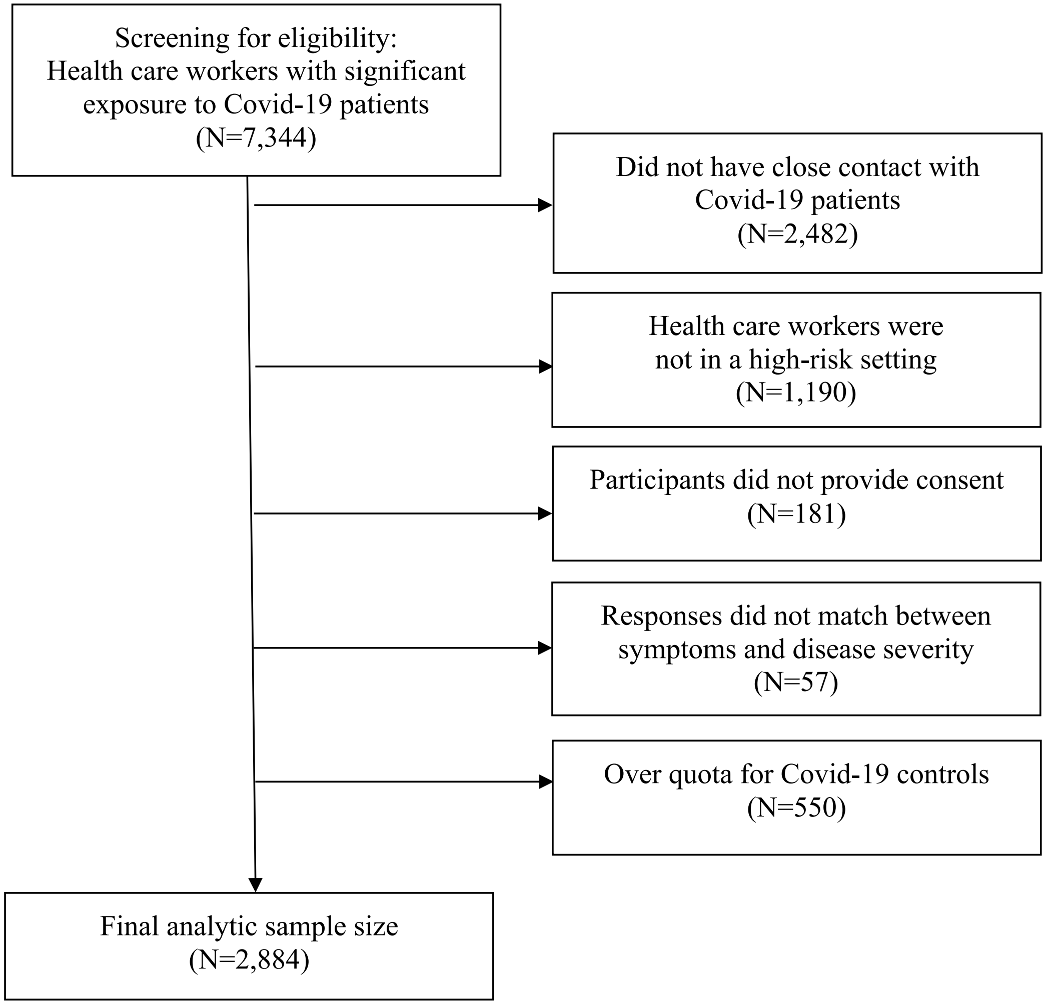

Supplement: Supplementary file 1 [file bmjnph-2021-000272supp001.pdf]
